# Effects of Heat Shock on Photosynthetic Properties, Antioxidant Enzyme Activity, and Downy Mildew of Cucumber (*Cucumis sativus* L.)

**DOI:** 10.1371/journal.pone.0152429

**Published:** 2016-04-11

**Authors:** Xiaotao Ding, Yuping Jiang, Ting Hao, Haijun Jin, Hongmei Zhang, Lizhong He, Qiang Zhou, Danfeng Huang, Dafeng Hui, Jizhu Yu

**Affiliations:** 1 Shanghai Key Lab of Protected Horticultural Technology, Horticultural Research Institute, Shanghai Academy of Agricultural Sciences, Shanghai 201106, China; 2 School of Agriculture and Biology, Shanghai Jiaotong University, Shanghai 202400, China; 3 Department of Biological Sciences, Tennessee State University, Nashville, Tennessee 37209, United States of America; Hainan University, CHINA

## Abstract

Heat shock is considered an abiotic stress for plant growth, but the effects of heat shock on physiological responses of cucumber plant leaves with and without downy mildew disease are still not clear. In this study, cucumber seedlings were exposed to heat shock in greenhouses, and the responses of photosynthetic properties, carbohydrate metabolism, antioxidant enzyme activity, osmolytes, and disease severity index of leaves with or without the downy mildew disease were measured. Results showed that heat shock significantly decreased the net photosynthetic rate, actual photochemical efficiency, photochemical quenching coefficient, and starch content. Heat shock caused an increase in the stomatal conductance, transpiration rate, antioxidant enzyme activities, total soluble sugar content, sucrose content, soluble protein content and proline content for both healthy leaves and downy mildew infected leaves. These results demonstrate that heat shock activated the transpiration pathway to protect the photosystem from damage due to excess energy in cucumber leaves. Potential resistance mechanisms of plants exposed to heat stress may involve higher osmotic regulation capacity related to an increase of total accumulations of soluble sugar, proline and soluble protein, as well as higher antioxidant enzymes activity in stressed leaves. Heat shock reduced downy mildew disease severity index by more than 50%, and clearly alleviated downy mildew development in the greenhouses. These findings indicate that cucumber may have a complex physiological change to resist short-term heat shock, and suppress the development of the downy mildew disease.

## Introduction

Heat shock is defined as the exposure of plants to a sudden and significant temperature increase for a short period of time [1]. As global temperature increases combined with the effects of more frequent extreme weather events, heat shock could have significant influences on plant physiology and development. During the heat shock, plants are subject to physical stress in their environment [2]. The initial effects of high temperature on plants include growth reduction, water loss, a change in photosynthetic efficiency, and oxidative stress [3]. Longer periods of high temperature stress may result in wilting, necrosis, leaf pigmentation loss for herbaceous plants, as well as leaf elongation repression [4]. Therefore, heat stress is considered as a problem for agriculture throughout the world [5, 6], and it may lead to dramatic losses in crop yields [7].

Photosynthesis is the most sensitive biological process influenced by high temperature stress [8, 9]. To protect the photosynthetic systems from injury, plants increase energy dissipation in PSII [10]. Heat stress has been reported to induce the overproduction of reactive oxygen species (ROS) [11], but plants may also activate antioxidant enzymes, such as superoxide dismutase (SOD) and ascorbate peroxidase (APX), to eliminate ROS or to alleviate the impact of ROS [12]. Other metabolites, including proline and soluble sugars that have been reported to be influenced by heat stress may also play an important role in the plant’s response to heat stress [13, 14]. So far, previous studies only investigated the impacts of heat stress on plant growth and physiological changes under ideal laboratory conditions [8, 12]. A more comprehensive study on plant photosynthetic response, antioxidant enzyme activities, and osmotic regulation in vegetable greenhouse condition is still lacking. While many studies reported the negative effects of heat stress [6, 15], a few studies reported the positive effects of heat shock against plant insect (whitefly [16] *Thrips tabaci* [17]), or disease (strawberry crown rot [18], pepper and tomato powdery mildew [19], and sweet basil downy mildew [20]). For example, Sato and Kubo [21] reported a practical use of heat shock to reduce the need for fungicide application in downy mildew infested cucumber while performing a simple test on some chlorophyll fluorescence parameters changes. Cucurbit downy mildew, caused by *Pseudoperonospora cubensis*, is a widespread disease affecting greenhouse and field-grown cucumbers and often leading to significant yield losses [22–24]. Although most cucurbits are susceptible to downy mildew [25], cucumber (*Cucumis sativus* L.) is the most susceptible plant. The use of chemicals such as fungicide becomes a limiting factor in the production of high quality and residue-free fruits and vegetables, especially for cucumbers [26, 27]. It would be beneficial to develop and test additional chemical-free methods such as heat shock treatment to improve disease resistance in crops.

In this study, we investigated the responses of physiological processes such as photosynthesis, chlorophyll fluorescence, carbohydrate metabolism, antioxidant enzyme activity and osmolytes to short-term heat shock in cucumber plants growing under normal greenhouse conditions as would occur in large-scale cucumber greenhouse production. Leaves with or without downy mildew were exposed to heat shock and a series of physiological properties and disease severity index were measured. The information generated from this study adds to our understanding of plant resistance to heat stress and for disease management.

## Materials and Methods

### Plant material and treatments

The cucumber variety used in this study (*Cucumis sativus* L. cv. Chunqiuwang No. 2) was selected by the Horticultural Research Institute of Shanghai Academy of Agricultural Sciences, China. Cucumber seeds were sown in a peat-vermiculite mixture (2:1, v/v) on February 10, 2013 and transplanted to greenhouses (40 m long and 8 m wide) at the two-leaf stage at a density of 3.2 plants m^-2^ on March 25. Four greenhouses were used in the experiment. Four blocks were implemented in each greenhouse with two hundred and fifty plants included in each block. A compound fertilizer(N-P_2_O_5_-K_2_O = 15-15-15) and an organic fertilizer (organic matter ≥ 30%) were applied as a basal dressing, and no additional fertilizer was applied. Plants were irrigated to ensure no water stress occurred during the entire experiment.

**Fig 1:**
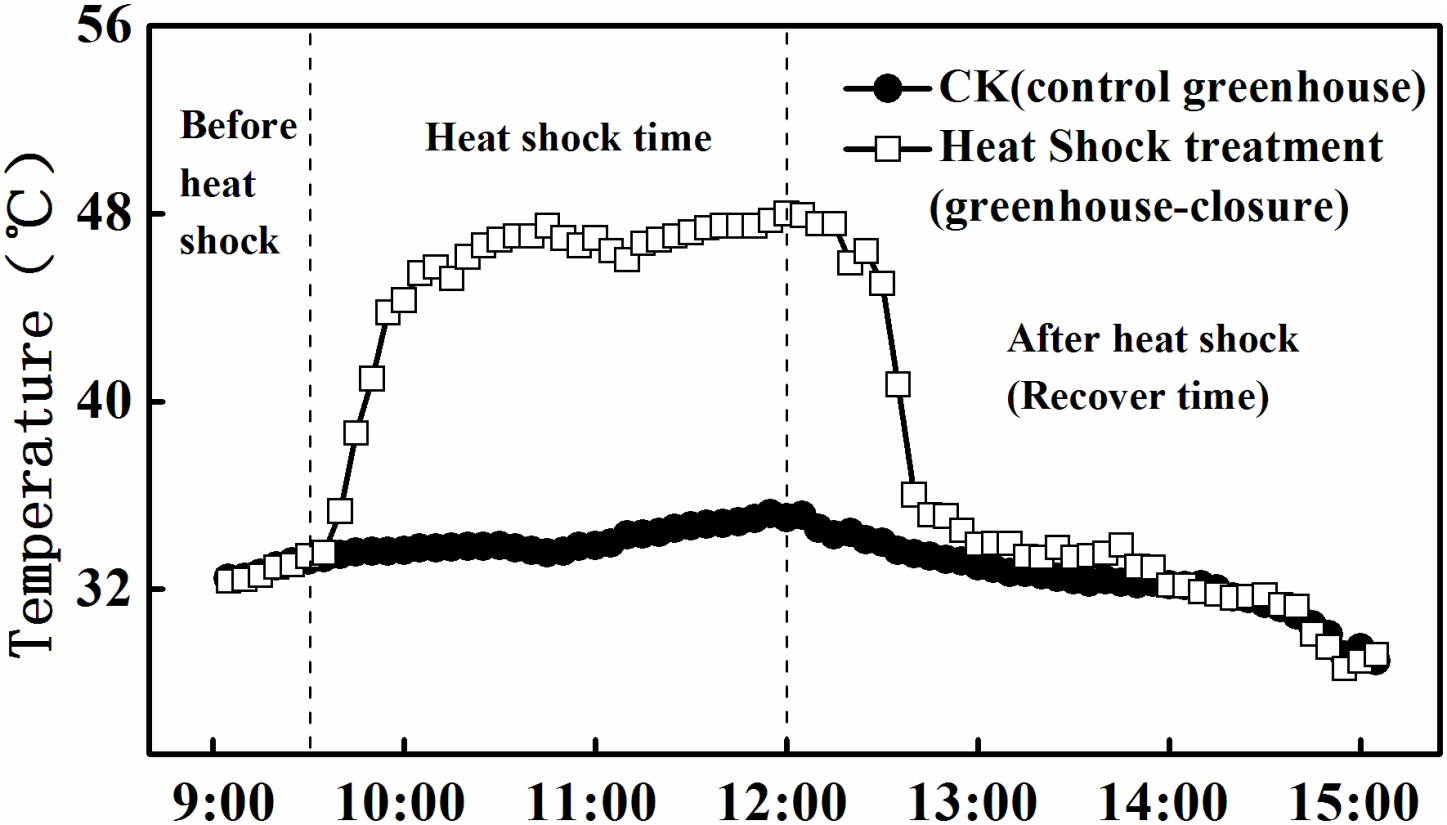
Temperature changes in heat shock treatment and control greenhouses.

The experiment was carried out when the cucumber plants possessed 20 leaves. A two-factor split plot design was used with the heat shock as main factor and downy mildew infection as the split factor. For heat shock treatment, two greenhouses were used and the other two were used under normal environmental conditions without the introduction of heat shock. Cucumber leaves were naturally infected with downy mildew under greenhouse conditions. Within each greenhouse, leaves were separated into downy mildew infected leaves (selected from the infected plants) and healthy leaves (selected from the healthy plants). Five leaves were selected from the downy mildew infected plants and healthy treatments in each block. Thus, four treatments in this experiment included: (1) healthy leaves without heat shock (“CK-HL”), (2) healthy leaves with heat shock (“HS-HL”), (3) downy mildew-infected leaves (selecting leaves at level 1: disease spot diameters < 0.5 cm) without heat shock (“CK-DML”), and (4) downy mildew-infected leaves (selecting leaves at level 1: disease spot diameters < 0.5 cm) with heat shock (“HS-DML”). For the heat shock treatment, the greenhouses were almost completely closed from 9:30 to 12:00 on a sunny day (May 25, 2013) to create a heat-stressed environment. After 12:00, the windows were gradually opened over a period of 30 minutes until they were completely open (at 12:30) and were kept open for the rest of the experiment. For the non-heat shock treatments, the windows of the greenhouses were kept open throughout the duration of the experiment. A temperature sensor was placed in the center of each greenhouse at a height of 1.5 m to record the temperature changes from 9:00 to 15:00 (Fig 1). We carefully checked the temperature sensor and slowly adjusted the window gap to make sure the highest temperature did not exceed 48°C in order to prevent irrevocable injury to leaves. Temperature in the heat shock greenhouses was sharply raised from 33°C at 9:30 to 45°C at 10:00, and increased to the maximum temperature of 48°C at 12:00, and decreased quickly to 33°C after the heat shock treatment was concluded. Temperature in the non-heat shock treatment greenhouses was relatively stable at 29–35°C and only increased slightly during a day.

### Leaf gas exchange and chlorophyll fluorescence analysis

Leaf gas exchange and chlorophyll fluorescence were measured at 9:30, 10:30, 12:00, and 14:00 on the day of treatment and at 10:30 on the first and second day after treatment. The biggest leaves of each treatment at same heights were harvested after each measurement, and the samples were frozen immediately in liquid nitrogen and stored at -80°C for further analysis.

Leaf gas exchange was measured with a Li-6400 Portable Photosynthesis System (Li-Cor Inc., Lincoln, NE, USA) on a fully developed leaf from the middle of each seedling. Irradiance level was set 600 μmol photons m^-2^ s^-1^. Air temperature, air relative humidity, and CO_2_ concentration were dependent on the greenhouse conditions. The CO_2_ assimilation rate or net photosynthetic rate (*P*_*n*_), stomatal conductance (*G*_s_) and transpiration rate (*T*_r_) of cucumber leaves were analyzed. Chlorophyll fluorescence was measured with a PAM-2100 Pulse Modulated Fluorometer (Walz, Effeltrich, Germany) on the same leaves as those used for the gas exchange measurements. For measuring steady-state fluorescence yield (Ft), an actinic light source (600 μmol photons m^-2^ s^-1^) was applied to achieve steady state photosynthesis, after which a second saturation pulse was applied for 0.7 s to obtain maximum fluorescence (F_m_’). The minimal fluorescence level in light-adapted state (F_o_’) was determined by illuminating the leaf with a 3 s far-red light. The actual photochemical efficiency of PSII (Ф_PSII_), and photochemical quenching coefficient (qP) were calculated as (Fm’–Ft)/ Fm’, (Fm’–Ft)/ (Fm’–Fo’), following Lu et al. [28]. Measurements of photosynthesis and fluorescence parameters were repeated once for each leaf, and five leaves in different plants were measured per block.

### Measurements of carbohydrates, proline and soluble protein

Leaf samples were freeze-dried and ground to determine the carbohydrate content. Total soluble sugars, sucrose, and starch were determined by using the method of Buysse and Merckx [29]. A 200 mg sample was extracted in 8 mL of 80% ethanol (80:20, v/v, ethanol/water) five times, centrifuged, and adjusted to 50 mL in volumetric flasks, and used for spectrophotometric determination of total soluble sugars (using 0.1 ml of extracted solution) and sucrose (using 0.4 ml of extracted solution). The pellets from centrifugation were oven-dried at 60°C for 48 h. Starch was measured by hydrolyzing the dried pellets for 3 h in 10 mL of 2% HCl at 100°C, centrifuging the extract, and adjusting the volume to 25 mL for spectrophotometric determination of the resultant sugars in the extract at 620 nm. For sucrose determination, sucrose was used for the standard curve, whereas for total sugar and starch determinations, glucose was used for the standard curves. Total soluble protein content was measured using Bradford reagent [30]. To determine the free proline level, 0.5 g of leaf sample was homogenized in 3% 5-sulfosalicylic acid, and the homogenate was filtered through filter paper [31]. The mixture was heated at 100°C for 1 h in a water bath after the addition of ninhydrin acid and glacial acetic acid. The reaction was then stopped in an ice bath. The mixture was extracted with toluene, and the absorbance of the fraction with toluene aspired from the liquid phase was read at 520 nm. The proline concentration was determined using a calibration curve [32].

### Antioxidant enzyme activity assay

For the enzyme assays, 0.3 g of leaf samples were ground in 3 mL of ice-cold 25 mM HEPES buffer (pH 7.8) containing 0.2 mM EDTA, 2 mM AsA, and 2% PVP. The homogenates were centrifuged at 4°C for 20 min at 12,000 *g*, and the supernatants were used for the determination of enzymatic activity. Superoxide dismutase (SOD) activity was measured in a reaction mixture containing 50 mM phosphate buffer (pH 7.8), 0.1 mM EDTA, 13 mM methionine, 75 μM nitroblue tetrazolium (NBT), 2 μM riboflavin, and 50 μl enzyme aliquot [33]. One unit of SOD activity was defined as the amount of enzyme required to cause a 50% inhibition of the rate of p-nitro blue tetrazolium chloride reduction at 560 nm. The method developed by Cakmak and Marschner [34], with some modifications, was used to determine the activity of guaiacol peroxidase (G-POD). The reaction mixture contained 25 mM phosphate buffer (pH 7.0), 0.05% guaiacol, 1.0 mM H_2_O_2_ and 100 μl enzyme extract. The increase in absorbance at 470 nm caused by guaiacol oxidation (E = 26.6 mM cm^-1^) was used to determine the G-POD activity. Catalase (CAT) was assayed as described by Durner and Klessing [35], and the activity was determined as a decrease in the absorbance at 240 nm for 1 min following the decomposition of H_2_O_2_. Ascorbate peroxidase (APX) was measured according to Nakano and Asada [36] by monitoring the rate of ascorbate oxidation at 290 nm. Data are expressed as specific activity with protein content determined using the method of Bradford [30].

### Disease assessment

Disease incidence and severity were assessed one day before the heat shock treatment, three days after the treatment, and two weeks after the treatment. Ten randomly selected plants in each treatment were examined for the presence or absence of disease symptoms and signs of pathogens. Disease severity was assessed using a visual estimate of symptoms for every leaf [24, 37]. Disease severity was categorized as follows: level 0: leaves presenting no symptoms of disease; level 1: disease spot diameters < 0.5 cm; level 2: 0.5 cm ≤ disease spot diameters ≤ 1 cm; and level 3: disease spot diameters > 1 cm. Downy mildew disease index (DMDI) = (∑different levels ×level leaf number) × 100 / (3 × total leaves). The assessment was replicated five times.

### Statistical analysis

Analysis of variance (ANOVA) was conducted on physiological variables and DMDI using the general linear model procedure (PROC GLM) of the Statistical Analysis System (SAS version 9.2, SAS Institute Inc. Cary, NC, USA). Mean values of treatments were estimated. Each value was presented as the mean ± standard deviation (SD) in figures, with a minimum of five replicates. Differences between treatment means of DMDI were tested using the Least Significant Difference (LSD) test at α = 0.05 level of significance. The data were plotted using Origin 7.0 software (Origin Lab, Northampton, MA, USA).

## Results

ANOVA of the effects of heat shock (HS), downy mildew (MW) and their interaction on physiological variables were performed (S1 Table). Results showed that all physiological variables were significantly influenced by the DM treatment before, during and after the heat shock treatment, except *G*_*s*_ at 10:30am and 2 d after heat shock, CAT activity and proline content at 9:30am, and at 12:00pm and 2:00pm. Significant effects of DM and HS interaction were found on *P*_*n*_, Ф_PSII_, starch content, total soluble sugar, CAT, proline content, and soluble protein content. During the early recovery period (2:00pm), HS treatment still had significant influences on most of the measured variables, but the effect disappeared mostly 2 d after the heat shock treatment. Only *T*_*r*_, Ф_PSII_, starch content, APX, G-POD, SOD, and soluble protein content were significantly influenced by the HS treatment.

### Leaf gas exchange and chlorophyll fluorescence analysis

**Fig 2:**
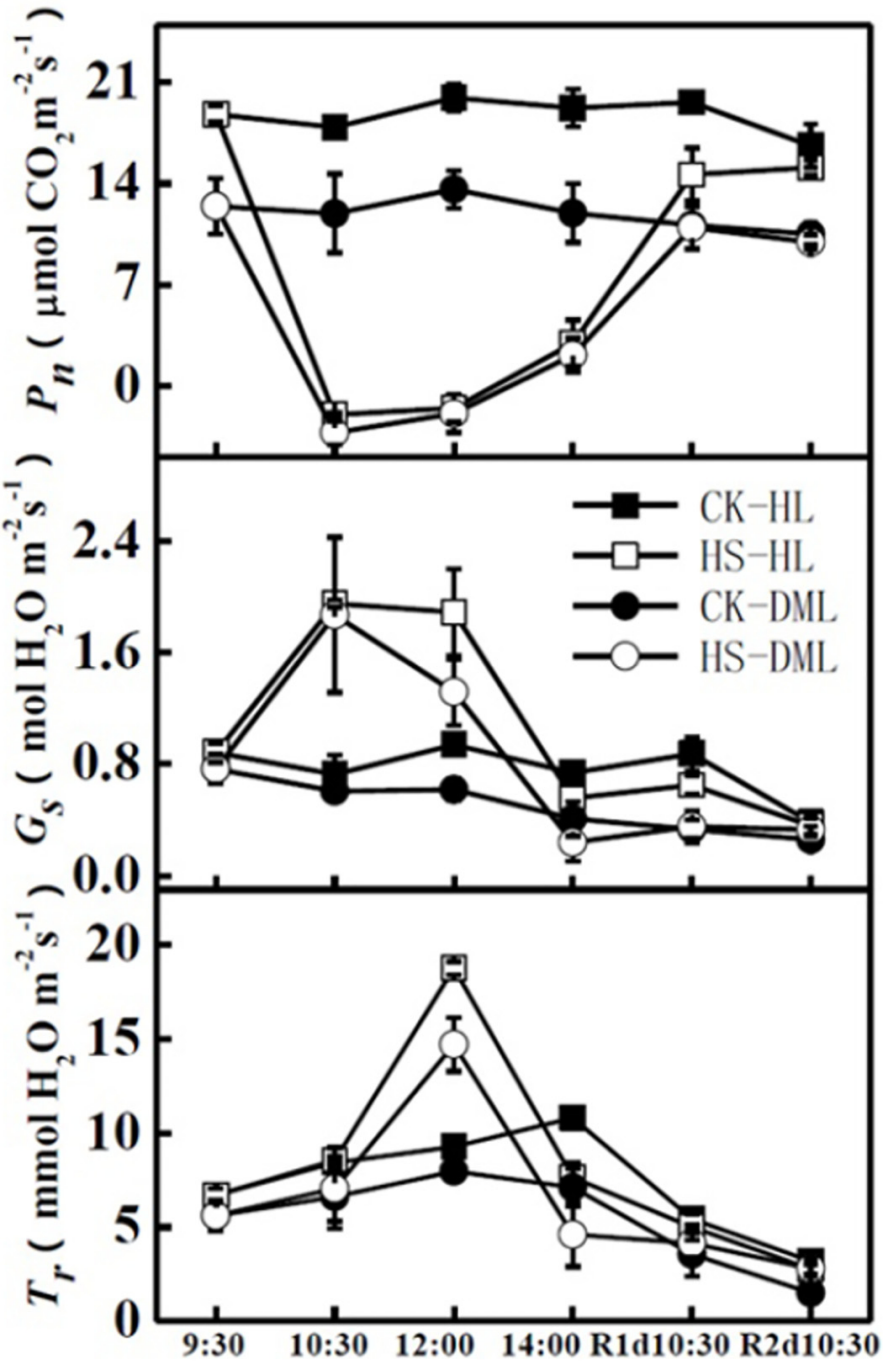
Effects of heat shock and recovery on the net photosynthetic rate (*P*_n_), transpiration rate (*T*_r_), and stomatal conductance (*G*_s_) in leaves of healthy and downy mildew infected cucumber. The data represent the mean ± SE (n = 5). CK-HL, healthy leaves without heat shock. HS-HL, healthy leaves with heat shock. CK-DML, downy mildew-infected leaves without heat shock. HS-DML, downy mildew-infected leaves with heat shock.

The *P*_n_, *G*_s_ and *T*_r_ were significantly influenced by the heat shock treatment (Fig 2). One hour after the heat shock treatment, *P*_*n*_ dramatically decreased, from 18.8 and 12.4 μmol CO_2_ m^-2^ s^-1^ at 9:30 to -2.0 and -3.3 μmol CO_2_ m^-2^ s^-1^ at 10:30, for healthy and downy mildew-infected leaves, respectively. This condition lasted for another 1.5 hours, until the windows of the greenhouse were opened at 12:00. After that, *P*_*n*_ gradually increased as the temperature in the greenhouse decreased. After one day, the *P*_*n*_ of HS-DML leaves recovered and was similar to the value of CK-DML, but *P*_*n*_ of HS-HL leaves was still lower than that of CK-HL. It took two days for the *P*_*n*_ of HS-HL leaves to recover to the level of CK-HL. The *P*_*n*_ of healthy leaves was higher than that of infected leaves under the non-heat shock treatment, but both of them showed similar response to heat shock treatment. In comparison to *P*_*n*_, *G*_s_ exhibited the reverse trend when leaves suffered heat stress. Leaf stomata opened prominently after a heat shock period of one hour; however, stomata gradually closed during the recovery period. In terms of transpiration, the *T*_r_ of both healthy leaves and downy mildew infected leaves showed a slight increase at 10:30 but peaked at 18.8 and 14.7 mmol H_2_O m^-2^ s^-1^, respectively, at 12:00. After that, the *T*_r_ gradually decreased to normal levels during the recovery period.

**Fig 3:**
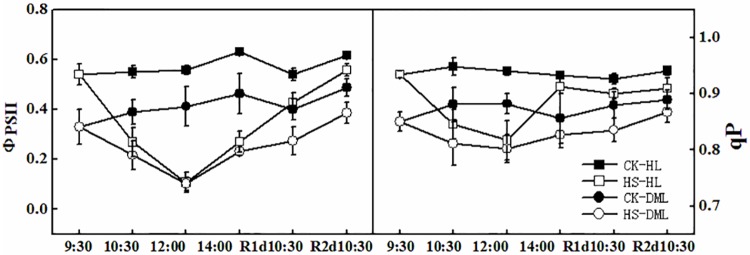
Effects of heat shock and recovery on the actual photochemical efficiency (Φ_PS II_), photochemical quenching coefficient (qP), apparent photosynthetic electron transport rate (ETR), and non-photochemical quenching coefficient (qN) in leaves of healthy and downy mildew-infected cucumber. The data represent the mean ± SE (n = 5).

Compared to leaves without infection, leaves that were infected with downy mildew had a lower Ф_PSII_ and qP (Fig 3). The high temperature caused a rapid decline of Ф_PSII_ and qP for both HS-HL and HS-DML treatments. In recovery time, Ф_PSII_ and qP slowly increased to approach the normal levels, and HS-HL recovered quicker than HS-DML treatments.

### Carbohydrate analysis

**Fig 4:**
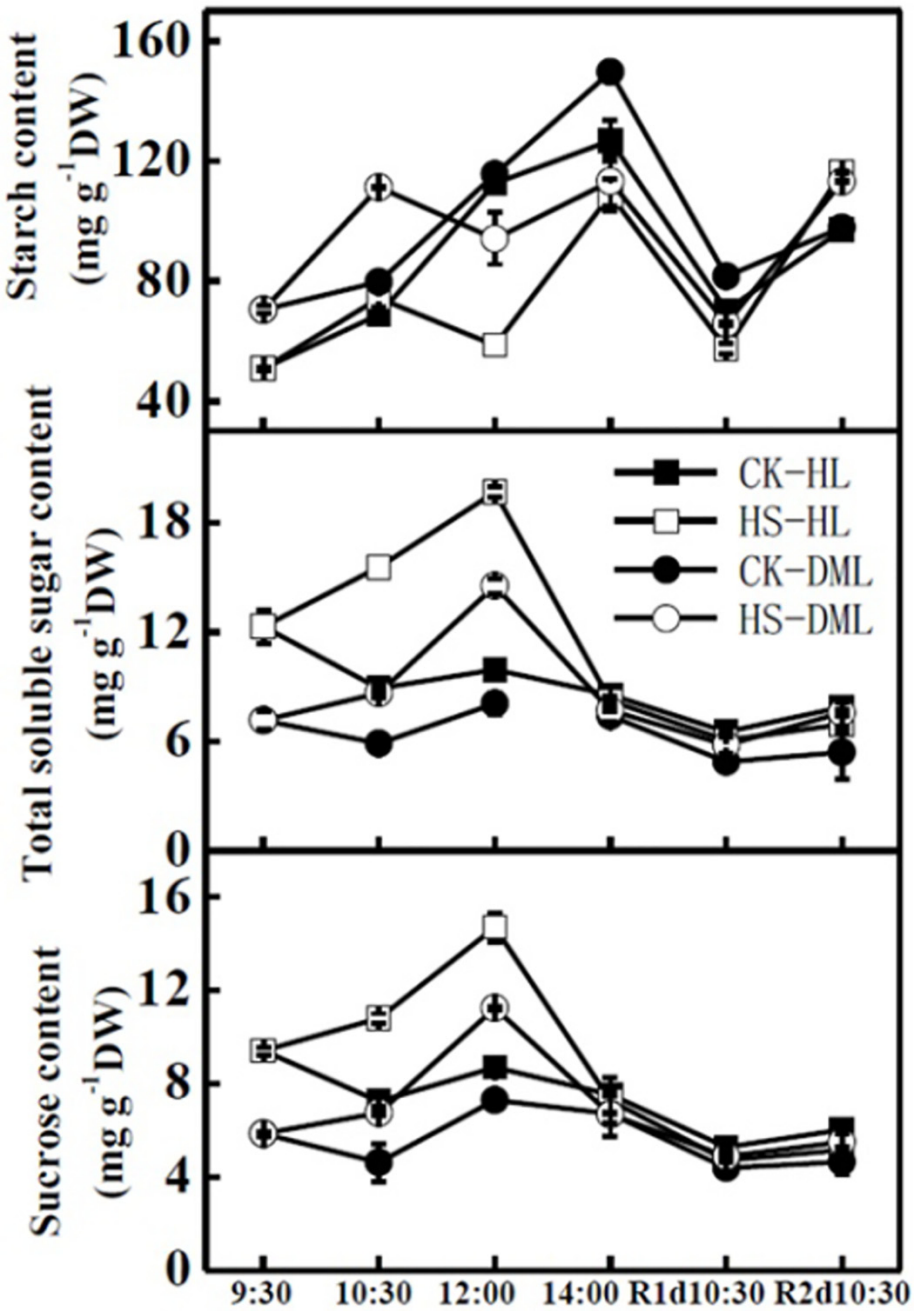
Effects of heat shock and recovery on the starch content, total soluble sugar content, and sucrose content in leaves of healthy and downy mildew-infected cucumber. The data represent the mean ± SE (n = 5).

Both the total soluble sugar content and the sucrose content of healthy leaves under normal temperatures were higher than those of downy mildew infected leaves (Fig 4). Under the heat shock treatment, the total soluble sugar content gradually increased from 12.3 (HL) and 7.2 (DML) mg g^-1^ DW at 9:30 to 19.7 (HS-HL) and 14.5 mg g^-1^ DW (HS-DML) at 12:00. Additionally, the sucrose content gradually increased from 9.4 (HL) and 5.9 (DML) mg g^-1^ DW at 9:30 to 14.7 (HS-HL) and 11.2 mg g^-1^ DW (HS-DML) at 12:00. By 14:00, the greenhouse temperature decreased to a normal level, and the total sugar and sucrose contents quickly decreased to the level of non-heat shock leaves, and remained at the same level as these CK leaves in the following recovery days. The starch content in the leaves gradually increased from the morning to the afternoon in the greenhouses under normal temperatures, and it was a little higher in DML leaves than in HL leaves. The starch content rose to 74.3 and 111.2 mg g^-1^ DW for HL and DML leaves, respectively, after heat shock for one hour, and then decreased notably at the end of the heat stress at 12:00. The starch content of HS leaves was lower than that of CK leaves on the first day of recovery but was higher on the following day.

### Antioxidant enzymes analysis

**Fig 5:**
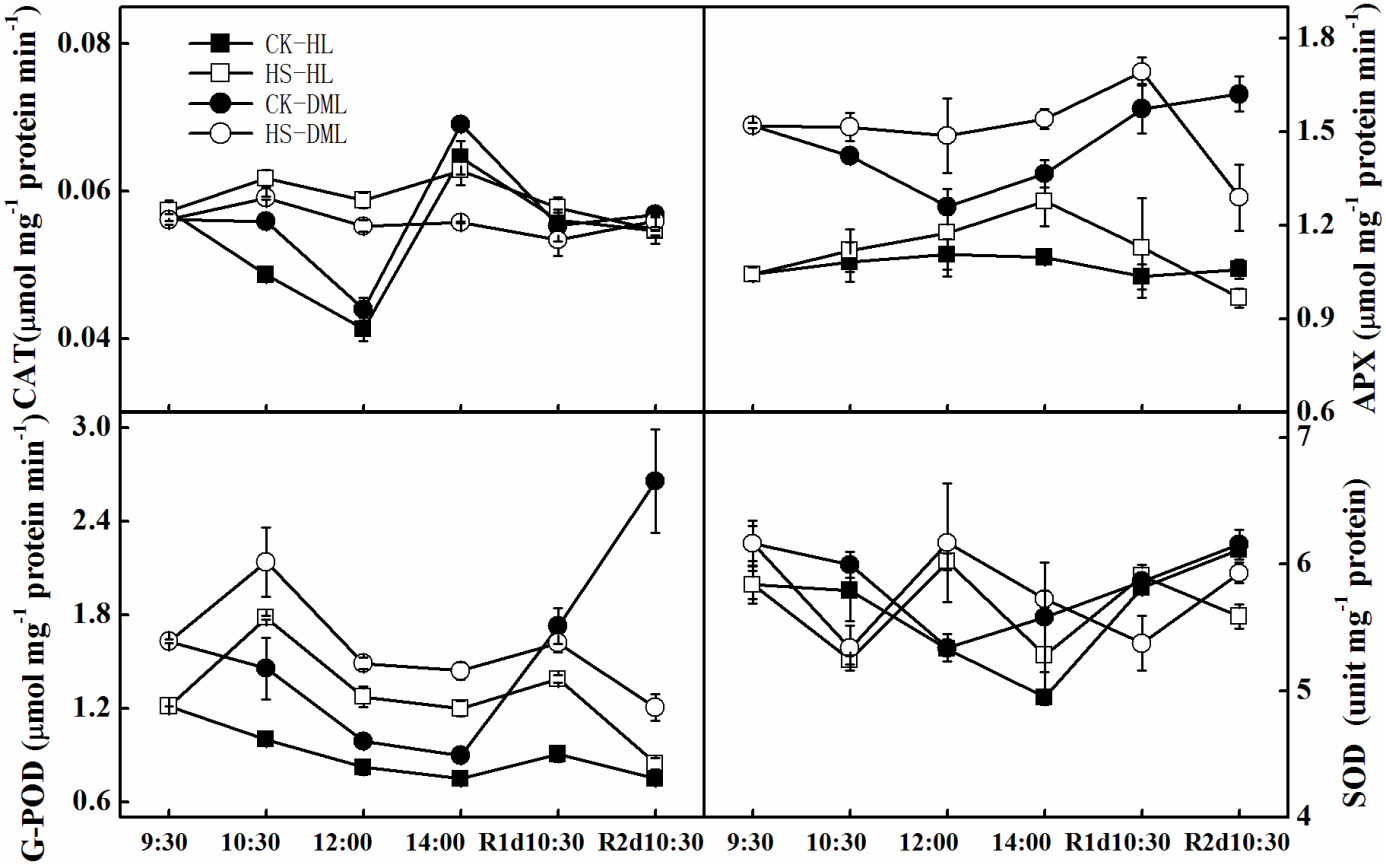
Effects of heat shock and recovery on catalase (CAT), ascorbate peroxidase (APX), superoxide dismutase (SOD) and guaiacol peroxidase (G-POD) activities in leaves of healthy and downy mildew-infected cucumber. The data represent the mean ± SE (n = 5).

Infection by downy mildew resulted in markedly enhanced enzyme activities of CAT, APX, G-POD and SOD (Fig 5). For both the healthy and DML leaves, enzyme activities of CAT, APX and G-POD had increased after 2.5 hours of the high temperature treatment, but SOD activities showed an initial decrease and a subsequent increase. After 2 hours of recovery (at 14:00), CAT activities in HS leaves had dropped slightly compared to leaves of CK, and activities of APX, G-POD, and SOD were still higher than in CK leaves. CAT activities of HS leaves gradually became similar to those of CK leaves after the following days of recovery. APX activities were still higher in HS leaves than CK leaves during the first recovery day but lower in the second recovery day. G-POD activities of CK-DML leaves were clearly enhanced in the recovery days, and G-POD activities of HS-HL leaves were always higher than CK-HL leaves, but lower than HS-DML leaves. SOD activities of HS leaves (both HL and DML) had, to some extent, lessened in the recovery days.

### Soluble protein content and proline content analysis

**Fig 6:**
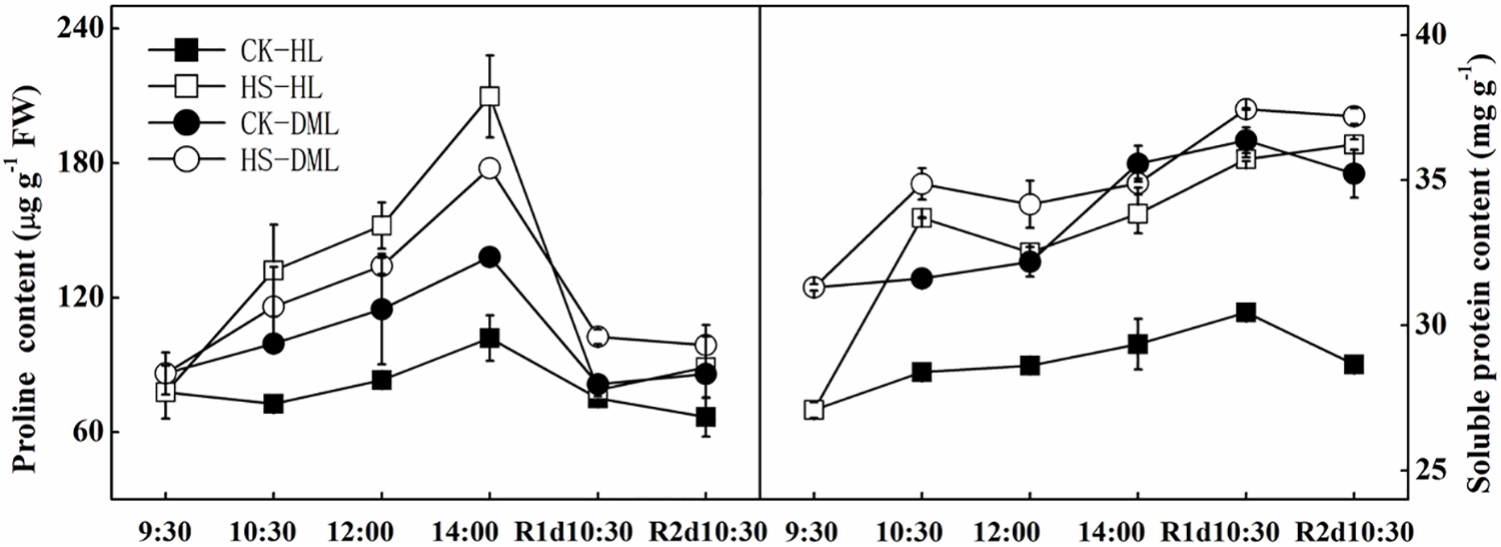
Effects of heat shock and recovery on the proline content and the soluble protein content in leaves of healthy and downy mildew-infected cucumber. The data represent the mean ± SE (n = 5).

The proline content and soluble protein content increased when leaves were infected with downy mildew (Fig 6). The proline content of HL leaves quickly increased as the high temperature treatment persisted and continued after the first 2 hours of recovery, and the proline content of HS-DML leaves also rose during that time, but less than that of HS-HL leaves. Although both dropped to normal levels in the following recovery days, HS-HL leaves exhibited a faster decrease than HS-DML leaves. HS-HL and HS-DML leaves maintained the high soluble protein content when suffering stress, and after stress, HS-DML leaves also had an increase in soluble protein content, but not as clearly as HS-HL leaves.

### Disease severity index analysis

**Fig 7:**
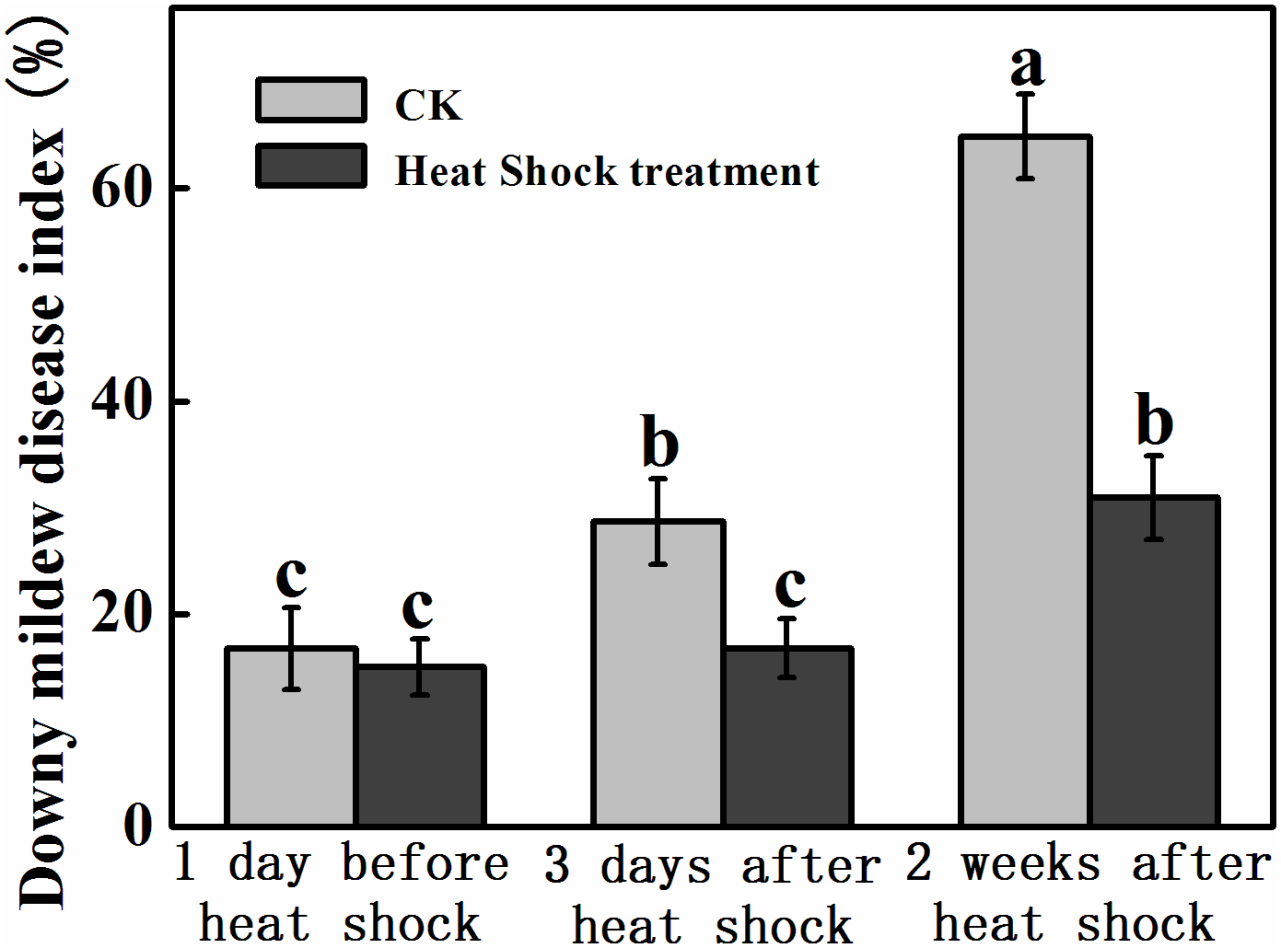
Effects of heat shock on downy mildew disease index of plants at different greenhouses. The data represent the mean ± SE (n = 5). Different letters indicate significant differences at P < 0.05 based on the Least Significant Difference test.

No significant difference in downy mildew disease indexes (DMDI) was observed for the infected plants in different greenhouses before the high temperature treatment (Fig 7). However, compared to the infected plants in the greenhouse maintained at normal temperatures, DMDI of plants in the heat shock greenhouse dropped 58.6% three days after the treatment. Notably, the heat shock treatment decreased DMDI by more than 50% two weeks later, with HS-DML and CK-DML plants developing DMDI of 31.0% and 64.9%, respectively.

## Discussion

Photosynthesis is the fundamental physiological process to provide energy and carbon assimilation for growth and reproduction of plants [38]. It is often inhibited before other cell functions are impaired due to its high sensitive to high temperature stress [9]. In the present study, the responses of the leaf photosynthetic parameters *P*_*n*_, *G*_s_ and *T*_r_ showed significant responses to the heat shock treatment. The *P*_n_ of both healthy and downy mildew infected leaves significantly decreased after the high temperature treatment, whereas *T*_r_ and *G*_s_ showed an opposite trend. These results indicate that when the cucumbers suffered from heat stress, the leaves opened their stomata to reduce leaf temperature through transpiration [39, 40]. Healthy leaves under the normal temperature clearly showed higher *P*_n_ than downy mildew infected leaves, which means that downy mildew disease reduced leaf photosynthesis under normal conditions but showed similar *P*_n_ responses to the heat shock treatment. Photosynthetic parameters generally reached normal levels in the subsequent recovery period indicating that short term heat shock may have limited and recoverable influences on plant physiology and growth.

Chlorophyll fluorescence is a very powerful tool to probe and elucidate the function of the photosynthetic apparatus, and its study is now widespread [41]. Photosystem II is considered to be one of the most thermo-sensitive components of the photosynthetic apparatus [42]. Ф_PSII_ was assessed from the quantum efficiency of photochemical energy dissipation which relates to the utilization of photons absorbed by the PSII antennae. qP was a measure of the proportion of PSII reaction centers capable of photochemistry and changes in qP are similar to the changes in Ф_PSII_ [43]. In the current study, Ф_PSII_ and qP plummeted during heat shock, which is consistent with the observation that the PSII reaction center and the light harvesting complexes are initially disrupted by high temperature [44]. This is probably due to the damage PSII sustains when exposed to cold [45] and heat [46] stress in higher plants. Ф_PSII_ and qP generally reached their normal levels in the recovery period, conforming chlorophyll fluorescence is a useful tool to assess the efficiency of the photosynthetic apparatus and the plant responses to the environment stress [47].

Carbohydrate metabolism is the major metabolic pathway in plants. Carbohydrate metabolism may change when environmental conditions change [48]. Sugar can control metabolism, growth and development, as well as biotic and abiotic stresses that alter sugar levels and modulate source-sink activities [49]. We found that both the total soluble sugar content and the sucrose content in leaves exposed to the normal temperature were higher than those in downy mildew infected leaves, while the starch content was lower. When cucumbers suffered heat shock, the leaves showed elevated total soluble sugar content and sucrose content and a reduction in starch. This indicates that starch breakdown and transformation to soluble carbohydrates occurred along with elevated energy production during heat stress [36, 50].

Antioxidative metabolism is involved in the defense response of plants. Antioxidant enzymes function as a defense system against deleterious free radicals in plant cells [51, 52]. The current study indicates that the activities of CAT, APX, G-POD and SOD evidently increased when plants were infected by downy mildew, which was consistent with the findings reported by Nostar *et al*. [32]. Oxidative stress is produced as a secondary stress during the heat stress response, which results in the abundant production of ROS [53]. We also found that the enzyme activities of CAT, APX, G-POD and SOD were enhanced under heat shock, suggesting that increased antioxidant activity in stressed tissues resulted in low levels of activated oxygen species, which might alleviate injury [54]. SOD, which causes dismutation of O_2_•^−^ to H_2_O_2_ and oxygen, was believed to form the first line of defense for removing ROS [55]. In addition to POD, CAT and APX scavenge H_2_O_2_ from cells [56]. Almeselmani et al. [11] and Dai et al. [15] also reported similar results, that the amelioration of high temperature stress induces oxidative stress by antioxidant enzymes. During the recovery period, the activities of APX and G-POD of CK-DML leaves had obviously increased, revealing that the downy mildew infected leaves became more and more seriously stressed.

Different plant responses, such as osmotic regulation, protein stabilization, hydroxyl radical scavenging and pH regulation were attributed to proline production when plants were subjected to abiotic stresses [57]. The accumulation of proline under stress in many plant species has been correlated with stress tolerance, with the concentration of proline shown to be generally higher in stress-tolerant plants than in stress-sensitive ones [58]. Many plants accumulate osmolytes such as proline, soluble sugars and soluble protein under environmental stresses [59]. In the current study, increased levels of proline and soluble proteins were observed in the heat shock-stressed cucumber leaves of both healthy and downy mildew infected leaves. The increased levels of proline and soluble proteins in the healthy leaf under the heat shock treatment were higher than those in the downy mildew infected leaves, which suggests that healthy leaves are more resistance to heat shock. Dai *et al*. [15] found that increased proline levels and soluble sugar levels in heat-stressed leaves enhanced the heat tolerance of cucumber seedlings. The accumulation of soluble protein, especially the heat shock proteins (HSPs) during heat shock, contributes to stress tolerance in plants [60]. Our results were also consistent with a more recent study by Li *et al*. [8] who concluded that protein protection plays an important role in maintaining high heat resistance in cucumbers grafted by luffa.

Disease incidence and severity are very important for the assessment of resistance to downy mildew in cucumbers under greenhouse conditions [24]. We compared the downy mildew disease index of cucumber plants with and without heat shock treatments and found that DMDI was markedly decreased at different times in the investigation in the heat shock greenhouse, which suggests that short term heat shock can restrain the spread of downy mildew disease and activate disease resistance in plants. Similar results were found by Sato and Kubo [21], who revealed that heat shock treatment assures stable growth and greatly reduces the amount of chemical protection needed for cucumbers. The main reason may be that spores in the leaves of infected plants lost infectivity in heat shock. Cohen and Rubin found that downy mildew mycelium inside leaves did not survive after exposure to at 45°C for several hours [20]. Powdery mildew in pepper and tomatoes and gray mold in sweet basil were also suppressed by daytime solar heat shock treatments [19].

This study demonstrated that heat shock may reduce the development of the downy mildew disease and have recoverable influences on cucumber physiology. But the heat shock treatment should be done carefully to make sure that temperatures do not exceed 48°C and plants need to be well watered to avoid heat damage. Plants may have several mechanisms to adapt to short term heat shock including: 1) enhanced thermal dissipation to protect leaves from damage caused by excessive energy excitation by increasing transpiration rates; 2) adjusted carbohydrate metabolism prompting starch breakdown and transformation to soluble carbohydrates; 3) increased antioxidant activity in stressed tissues resulting in low levels of activated oxygen species to alleviate injury; and 4) accumulation of osmolytes such as proline and soluble sugars to stabilize the structure of macromolecule, decrease the cellular acidification, and elevate resistance ability. Healthy leaves showed more adaptation ability to heat shock stress than downy mildew-infected leaves.

In conclusion, this study demonstrated that cucumber can adjust photosynthesis, carbohydrate content, antioxidative metabolism and osmolyte accumulation to adapt to short-term heat shock and suppress the spread of downy mildew disease. Heat shock may be used as a simple and environmentally friendly method to improve disease resistance and agricultural production of cucumber in greenhouses.

## Supporting Information

S1 Table ANOVA of the effects of heat shock (HS), downy mildew (MW) and their interaction on physiological variables. (DOC)
