# Supplementary material for: Effects of Heat Shock on Photosynthetic Properties, Antioxidant Enzyme Activity, and Downy Mildew of Cucumber (Cucumis sativus L.)
Source: PLoS One. 2016 Apr 11;11(4):e0152429. doi: 10.1371/journal.pone.0152429 (PMC4827809; doi:10.1371/journal.pone.0152429)
Supplement: S1 Table — (DOC) [file pone.0152429.s001.doc]

S1 Table. ANOVA of the effects of heat shock (HS), downy mildew (MW) and their interaction on physiological variables

| Time | Treatment | | | *P_n_* | *G_s_* | *T_r_* | | PSII | | qP | | Starch content | | Total soluble sugar | | Sucrose content |
| --- | --- | --- | --- | --- | --- | --- | --- | --- | --- | --- | --- | --- | --- | --- | --- | --- |
| 9:30AM | DM | | | 143.19^**^ | 7.17^*^ | 13.23^**^ | | 77.33^**^ | | 50.81^**^ | | 1097.46^**^ | | 139.61^**^ | | 2171.88^**^ |
| 10:30AM | HS | | | 583.81^**^ | 91.68^**^ | 0.22 | | 105.72^**^ | | 9.14^**^ | | 264.99^**^ | | 1130.53^**^ | | 86.27^**^ |
|  | DM | | | 24.70^**^ | 0.6 | 7.56^*^ | | 23.37^**^ | | 3.10^△^ | | 442.54^**^ | | 1253.87^**^ | | 118.94^**^ |
|  | HS*DM | | | 10.59^**^ | 0.02 | 0.07 | | 5.99^*^ | | 0.31 | | 130.80^**^ | | 194.13^**^ | | 5.46^△^ |
| 12:00PM | HS | | | 1272.19^**^ | 65.41^**^ | 631.94^**^ | | 319.78^**^ | | 18.81^**^ | | 99.9^**^ | | 855.90^**^ | | 505.74^**^ |
|  | DM | | | 41.61^**^ | 19.22^**^ | 69.00^**^ | | 12.52^**^ | | 2.43 | | 26.12^**^ | | 159.20^**^ | | 120.46^**^ |
|  | HS*DM | | | 33.02^**^ | 1.52 | 18.05^**^ | | 10.72^**^ | | 0.86 | | 18.53^**^ | | 35.25^**^ | | 21.90^**^ |
| 2:00PM | HS | | | 296.94^**^ | 8.11^*^ | 33.83^**^ | | 196.23^**^ | | 0.88 | | 29.54^**^ | | 0.01 | | 0.09 |
|  | DM | | | 28.71^**^ | 27.51^**^ | 47.20^**^ | | 24.45^**^ | | 9.54^**^ | | 7.41^*^ | | 13.60^*^ | | 2.08 |
|  | HS*DM | | | 18.17^**^ | 0.01 | 0.43 | | 9.47^**^ | | 0.03 | | 3.35 | | 1.92 | | 0.05 |
| 10:30am 1 d after heat shock treatment | HS | | | 14.34^**^ | 4.91^*^ | 0.04 | | 41.01^**^ | | 4.58^*^ | | 317.90^**^ | | 0.7 | | 2.53 |
|  | DM | | | 78.93^**^ | 82.57^**^ | 14.03^**^ | | 62.58^**^ | | 11.28^**^ | | 150.35^**^ | | 11.94^*^ | | 7.74^*^ |
|  | HS*DM | | | 12.53^**^ | 6.77^*^ | 1.96 | | 0.18 | | 0.29 | | 3.37 | | 5.94^*^ | | 0.21 |
| 10:30am 2 d after heat shock treatment | HS | | | 1.07 | 0.11 | 8.50^*^ | | 34.85^**^ | | 2.76 | | 2284.37^**^ | | 1.98 | | 0 |
|  | DM | | | 142.93^**^ | 2.34 | 30.92^**^ | | 120.64^**^ | | 8.36^*^ | | 9.93^*^ | | 4.38^△^ | | 2.64 |
|  | HS*DM | | | 3.51^△^ | 2.95 | 32.39^**^ | | 2.38 | | 0.09 | | 30.41^**^ | | 11.59^**^ | | 7.13^*^ |
| Time | | Treatment | CAT | | APX | | G-POD | | SOD | | Proline content | | Soluble protein content | |  | |
| 9:30AM | | DM | 2.97 | | 2836.43^**^ | | 6314.10^**^ | | 18.40^**^ | | 1.85 | | 1573.16^**^ | |  | |
| 10:30AM | | HS | 200.94^**^ | | 4.75^△^ | | 83.99^**^ | | 29.29^**^ | | 17.94^**^ | | 669.22^**^ | |  | |
|  |  | DM | 16.61^**^ | | 152.83^**^ | | 25.92^**^ | | 1.79 | | 0.36 | | 176.30^**^ | |  | |
|  |  | HS*DM | 73.73^**^ | | 0.88 | | 0.39 | | 0.24 | | 5.8^*^ | | 38.75^**^ | |  | |
| 12:00PM | | HS | 348.71^**^ | | 7.71^*^ | | 207.34^**^ | | 17.93^**^ | | 19.45^**^ | | 106.73^**^ | |  | |
|  |  | DM | 0.27 | | 18.81^**^ | | 33.79^**^ | | 0.19 | | 0.46 | | 85.12^**^ | |  | |
|  |  | HS*DM | 16.16^**^ | | 2.19 | | 0.51 | | 0.15 | | 6.18^*^ | | 11.36^**^ | |  | |
| 2:00PM | | HS | 51.42^**^ | | 40.56^**^ | | 396.7^**^ | | 1.65 | | 74.94^**^ | | 27.21^**^ | |  | |
|  |  | DM | 1.44 | | 91.20^**^ | | 62.07^**^ | | 8.48^*^ | | 0.06 | | 98.58^**^ | |  | |
|  |  | HS*DM | 30.81^**^ | | 0 | | 3.39 | | 0.26 | | 16.09^*^ | | 49.52^**^ | |  | |
| 10:30am 1 d after heat shock treatment | | HS | 0.02 | | 3.92^△^ | | 40.32^**^ | | 4.15^△^ | | 28.35^**^ | | 586.36^**^ | |  | |
|  |  | DM | 6.23^*^ | | 105.74^**^ | | 317.85^**^ | | 5.91^△^ | | 41.03^**^ | | 851.31^**^ | |  | |
|  |  | HS*DM | 3.04 | | 0.06 | | 99.27^**^ | | 8.81^*^ | | 14.01^*^ | | 256.75^**^ | |  | |
| 10:30am 2 d after heat shock treatment | | HS | 0.3 | | 35.73^**^ | | 308.35^**^ | | 36.28^**^ | | 6.78^△^ | | 336.93^**^ | |  | |
|  |  | DM | 5.96^*^ | | 155.75^**^ | | 864.67^**^ | | 9.59^*^ | | 4.71^△^ | | 209.40^**^ | |  | |
|  |  | HS*DM | 0.48 | | 11.47^**^ | | 403.19^**^ | | 5.81^△^ | | 0.48 | | 115.27^**^ | |  | |

Numbers are F values. *P_n_*: net photosynthetic rate, *G_s_*: stomatal conductance, *T_r_*: transpiration, PSII: actual photochemical efficiency of PSII, qP: photochemical quenching coefficient, CAT: catalase, APX: ascorbate peroxidase, G-POD, guaiacol peroxidase, SOD: superoxide dismutase. *: significant at α=0.05; **: significant at α=0.01; △: significant at α =0.10.
